# Estimating the distribution of *Oryzomys palustris*, a potential key host in expanding rickettsial tick-borne disease risk

**DOI:** 10.1002/ecs2.4445

**Published:** 2023-03-15

**Authors:** Catherine A. Lippi, Samuel Canfield, Christina Espada, Holly D. Gaff, Sadie J. Ryan

**Affiliations:** 1 Department of Geography and Emerging Pathogens Institute, University of Florida, Gainesville, Florida, USA; 2 Department of Biology, Old Dominion University, Norfolk, Virginia, USA

**Keywords:** *Amblyomma maculatum*, *Oryzomys*, rickettsia, species distribution model, tick-borne diseases

## Abstract

Increasingly, geographic approaches to assessing the risk of tick-borne diseases are being used to inform public health decision-making and surveillance efforts. The distributions of key tick species of medical importance are often modeled as a function of environmental factors, using niche modeling approaches to capture habitat suitability. However, this is often disconnected from the potential distribution of key host species, which may play an important role in the actual transmission cycle and risk potential in expanding tick-borne disease risk. Using species distribution modeling, we explore the potential geographic range of *Oryzomys palustris*, the marsh rice rat, which has been implicated as a potential reservoir host of *Rickettsia parkeri*, a pathogen transmitted by the Gulf Coast tick (*Amblyomma maculatum*) in the southeastern United States. Due to recent taxonomic reclassification of *O. palustris* subspecies, we reclassified geolocated collections records into the newer clade definitions. We modeled the distribution of the two updated clades in the region, establishing for the first time, range maps and distributions of these two clades. The predicted distribution of both clades indicates a largely Gulf and southeastern coastal distribution. Estimated suitable habitat for *O. palustris* extends into the southern portion of the Mid-Atlantic region, with a discontinuous, limited area of suitability in coastal California. Broader distribution predictions suggest potential incursions along the Mississippi River. We found considerable overlap of predicted *O. palustris* ranges with the distribution of *A. maculatum*, indicating the potential need for extended surveillance efforts in those overlapping areas and attention to the role of hosts in transmission cycles.

## INTRODUCTION

Zoonotic vector-borne disease systems are ecologically complex due to the numerous interactions between pathogens, vectors, and hosts, in the context of the environment, that ultimately determine the risk of disease in humans. The presence of competent arthropod vectors is sometimes used as a proxy for pathogen exposure and transmission risk, particularly when transmission cycles and other risk factors are poorly understood or unknown (Lippi, Gaff, White, & Ryan, 2021; Lippi, Ryan, et al., 2021). This is the case for many tick-borne pathogen transmission systems, where there can be major knowledge gaps concerning wildlife reservoirs, environmental associations, or even the etiology of disease.

Leveraging tick presence to estimate geographic risk has logistical advantages, as existing data sources (e.g., surveillance programs, museum collections, online data repositories) can provide ample occurrence points for spatial risk modeling and are typically easier to obtain than protected human health data. Nevertheless, vector presence alone is not sufficient to maintain transmission cycles. Many medically important ticks are often habitat generalists, with broad geographic distributions exceeding known disease risk areas (Lippi, Gaff, White, St. John, et al., 2021). Additionally, ticks with expanding ranges can be associated with increases in the burden and diversity of pathogens in newly established locations compared with their historic range (Fornadel et al., 2011; Nadolny & Gaff, 2018; Ogden et al., 2013). These instances highlight the need to understand other components of transmission cycles that drive the risk of exposure beyond vector presence. Reservoir hosts are an important, yet in many instances underexplored, component of zoonotic tick-borne transmission cycles (Tomassone et al., 2018). When considering geographic risks of pathogen transmission, the distribution of wildlife hosts may serve as another important proxy for exposure risk. Therefore, while the use of ticks as proxies of exposure may be advisable in the absence of additional data, determining the distributions of reservoir hosts needed for pathogen amplification and spillover may serve as more nuanced indicators of risk and provide a focus for public health messaging and resources.

Tick-borne diseases have garnered increased attention from the medical community and public health practitioners over the past decade (Lippi, Ryan, et al., 2021; Rochlin & Toledo, 2020). This is largely a result of the increasing incidence of diseases such as Lyme borreliosis and spotted fever group (SFG) rickettsioses (CDC, 2021). Tick-borne infections are often underdiagnosed and go unreported, in part due to nondescript clinical presentations (e.g., febrile illness) and a lack of accurate diagnostic testing (Madison-Antenucci et al., 2020; Rosenberg et al., 2018). Therefore, public health advisories aimed at preventing exposure to potentially infective tick bites are an important aspect of managing these diseases. The Gulf Coast tick (*Amblyomma maculatum*) is a species of medical and veterinary importance in the Americas (Paddock & Goddard, 2015). *Amblyomma maculatum* is a competent vector of *Rickettsia parkeri*, the causative agent of *Rickettsia parkeri* rickettsiosis, a SFG disease (Paddock et al., 2004; Paddock & Goddard, 2015). *Amblyomma maculatum* is a habitat specialist found in open fields dominated by grasses and shrubs, requiring high rainfall, temperature, and humidity (Nadolny & Gaff, 2018; Paddock & Goddard, 2015). In the United States, *A. maculatum* has been undergoing a northward range expansion from its historic range along the Gulf Coast, most recently establishing populations in Connecticut, Illinois, and New York (Molaei et al., 2021; Phillips et al., 2020; Ramírez-Garofalo et al., 2022). Importantly, studies have shown that the prevalence of *R. parkeri* infections is higher in *A. maculatum* collected from some newly established locations, a possible result of higher pathogen burden in new vertebrate host populations (Fornadel et al., 2011; Nadolny & Gaff, 2018). Therefore, identifying competent reservoir hosts that underpin transmission cycles and delineating their distributions could provide better estimates of risk in the face of vector range expansion.

The marsh rice rat (*Oryzomys palustris*) is a suspected reservoir host for *R. parkeri* (Cumbie et al., 2020) and a vertebrate host for *A. maculatum* (Nadolny & Gaff, 2018; Wilson & Durden, 2003). Recent field studies have indicated that *O. palustris* may be involved in the pathogen transmission cycle of *R. parkeri*; the rats themselves have tested positive for infection, and *A. maculatum* collected from rats in the field have also tested positive for the pathogen (Cumbie et al., 2020). The distribution of *O. palustris* has been previously described with observation-based range maps, which are limited to the extent of field observations (Wolfe, 1982). Coarse species range maps are useful in certain contexts but are prone to localized errors and provide no information on spatial variation in the probability of species occurrences (Maréchaux et al., 2017). Further, recently proposed taxonomic changes to the *O. palustris* species complex are relevant to our understanding of where potential reservoirs are located, yet are available only as range maps (Hanson et al., 2010). To our knowledge, species distribution models (SDMs) have not been used to estimate the suitable geographic range of marsh rice rats. Frequently used in ecology and conservation research, SDMs have become an increasingly utilized methodology in medical geography and public health for estimating the geographic distribution of vector species. A typical SDM workflow entails using occurrence records for the species of interest with environmental predictor layers as inputs for an SDM algorithm, which produces spatially continuous outputs of potential habitat suitability throughout the study area. Here, we apply this technique to estimate the geographic range of the marsh rice rat to assess its potential role in tick-borne pathogen transmission.

## METHODS

### Occurrence data

**FIGURE 1:**
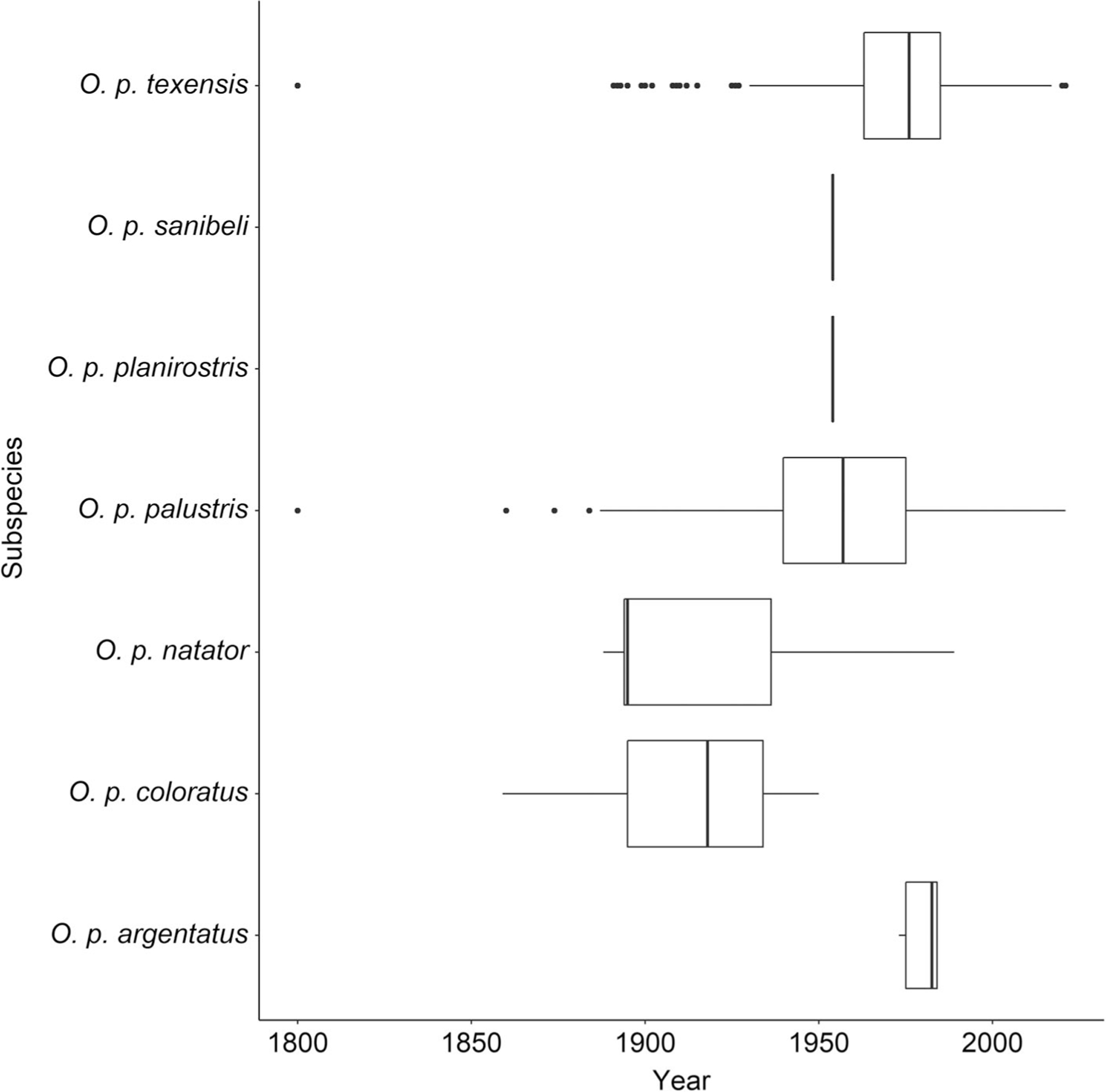
Collection record date ranges for Global Biodiversity Information Facility records of *Oryzomys palustris* subspecies used in this study. Boxplots depict the distribution of collection record totals for each subspecies (i.e., minimum, mean, and maximum values, with outliers shown as points).

Occurrence records for *O. palustris* were downloaded from the Global Biodiversity Information Facility (GBIF) (www.gbif.org), an international network that collates museum records and information on where and when species were collected. The collection timeframe for GBIF records extended from 1800 to 2021, though most occurrences were collected between 1888 and 1999, with few records outside this range (Figure 1). The full dataset (*n* = 7276) included many records that had not been georeferenced (*n* = 2457) (i.e., collection records with no associated latitude/longitude coordinates). The GEOLocate platform (www.geo-locate.org) was used to recover positional coordinates for records that had not been georeferenced but had locality data (i.e., a text description of the collection site was available) associated with records (*n* = 1727) (Murphey et al., 2004). Returned point locations with low precision, where locality data could be matched to several potential sites, were not retained for model building. The spatial uncertainty of georeferenced data on GBIF and coordinates recovered with GEOLocate varied substantially, and we excluded records where spatial uncertainty exceeded 10 km to maintain comparable resolution with environmental covariates. A total of 819 locations were recovered using the GEOLocate workflow.

**FIGURE 2:**
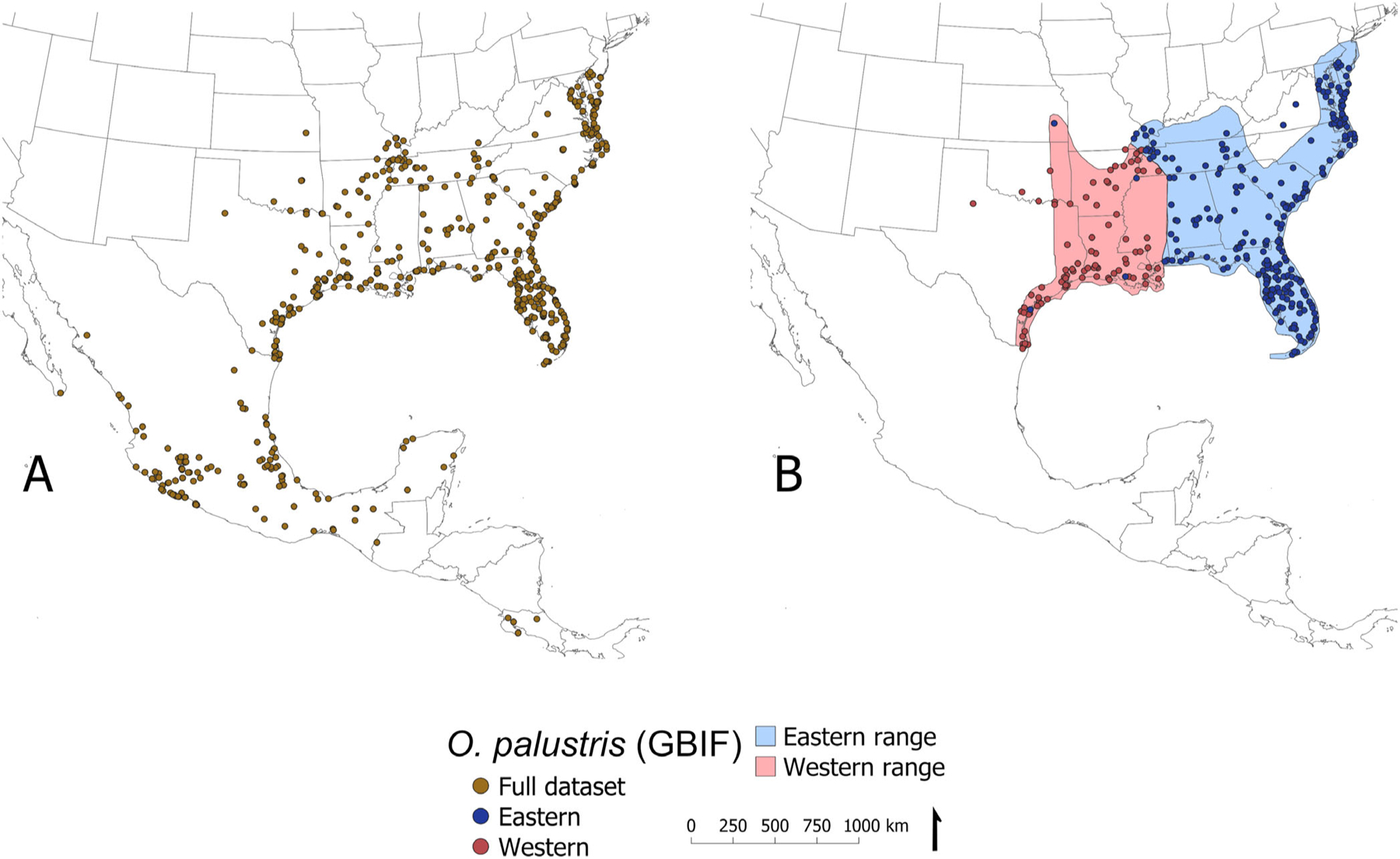
(A) Map of the United States and Central America and the spatial distribution of Global Biodiversity Information Facility (GBIF) specimen data records recognized as *Oryzomys palustris*. (B) A 10-km spatial thinning of the eastern and western clades, following Hanson et al. (2010).

Morphological and molecular evaluations of the *O. palustris* species complex have led to proposed taxonomic revisions over the past decade. Notably, Hanson et al. (2010) conducted a taxonomic assessment of subspecies in the *O. palustris* and *O. couesi* species complexes, utilizing three genetic markers as lines of evidence. The subspecies examined within the *O. palustris* clade were *O. p. texensis*, *O. p. palustris*, *O. p. coloratus*, *O. p. planirostris*, *O. p. sanibeli*, and *O. p. argentatus* (synonymous with *O. p. natator*; Baker et al., 2003; Humphrey & Setzer, 1989). Two clades within the *O. palustris* group showed considerable genetic divergence: a western clade consisting largely of *O. p. texensis* and an eastern clade comprising *Oryzomys palustris* and the remaining subspecies. Though Hanson et al. support the elevation of *O. p. texensis* to species following the Genetic Species Concept, these proposed taxonomic updates were not reflected in data obtained from GBIF, particularly for older collection events. Therefore, we created three separate spatial datasets of marsh rice rat occurrences for model building: (1) all geolocated GBIF records identified as *O. palustris* (*n* = 2195), plus two subsets derived according to clades identified in Hanson et al. (2010); (2) the eastern *O. palustris* clade; and (3) the western *O. p. texensis* clade. The full dataset of *O. palustris* occurrences was partitioned into subsets by combining records with valid subspecies provided and spatial assignation of records without subspecific epithets. To conduct this spatial assignation, we rendered the published images of the geographic distribution of *O. palustris* and *O. p. texensis* as spatial layers, using QGIS (ver 3.24.0), and georeferenced the collection points, yielding 1251 and 570 occurrence locations, respectively (Figure 2) (Hanson et al., 2010).

To prepare the geospatial occurrence data for model input, occurrence records in each of the three datasets were spatially thinned at a 10-km radius using spThin in R (Aiello-Lammens et al., 2015), removing duplicates and retaining one presence point per predictor pixel at the resolution of the study. The final datasets for model building included 336 occurrences in the full *O. palustris* dataset, 249 occurrences in the eastern dataset, and 90 occurrences in the western dataset. Each dataset was randomly partitioned into a 75% training/25% testing split for calculation of external model accuracy metrics.

### Environmental data

Environmental factors that have broad biological implications for landscape-level habitat suitability of small mammals were considered for initial model development, including indicators of precipitation, temperature, elevation, and soil conditions. A total of 22 environmental covariates at 5 min (~10 km^2^) spatial resolution were selected for variable screening (Table 1). Long-term climate data were obtained from WorldClim (ver 1.4) (Hijmans et al., 2005), which included 19 bioclimatic variables for monthly temperature and rainfall values with biologically derived products to represent annual trends, seasonality, and extreme or limiting environmental factors. WorldClim 1.4 incorporates climate averages from the years 1960–1990 and was chosen to align with the species occurrence data obtained for this study. Bioclimatic variables considered for model building included Annual Mean Temperature (Bio1), Mean Diurnal Range (Bio2), Isothermality (Bio3), Temperature Seasonality (Bio4), Max Temperature of Warmest Month (Bio5), Min Temperature of Coldest Month (Bio6), Temperature Annual Range (Bio7), Mean Temperature of Wettest Quarter (Bio8), Mean Temperature of Driest Quarter (Bio9), Mean Temperature of Warmest Quarter (Bio10), Mean Temperature of Coldest Quarter (Bio11), Annual Precipitation (Bio12), Precipitation of Wettest Month (Bio13), Precipitation of Driest Month (Bio14), Precipitation Seasonality (Bio15), Precipitation of Wettest Quarter (Bio16), Precipitation of Driest Quarter (Bio17), Precipitation of Warmest Quarter (Bio18), and Precipitation of Coldest Quarter (Bio19). Gridded elevation data were also obtained from WorldClim (ver 2.1) (Fick & Hijmans, 2017), derived from the Shuttle Radar Topography Mission (SRTM). Soil characteristics considered for model building were acquired from the International Soil Reference and Information Centre (ISRIC) and included organic carbon stock at a 30-cm depth (Hengl et al., 2017) and volumetric water content at 33 kPa in 0–5 cm range (Poggio et al., 2021). The timeframe during which most records were collected precluded the use of other descriptive land cover products in our analyses, as these products did not align with the collection period of most records, potentially misrepresenting historically suitable habitats.

Multicollinearity in environmental covariates can lead to known issues in SDMs, such as distortion of estimated effects of predictors (Dormann et al., 2013). This is a standard concern when modeling with numerous variables, particularly those derived from similar variables (e.g., bioclimatic variables derived from WorldClim data). While some SDM algorithms, such as boosted regression trees (BRTs), exhibit moderate resilience to predictor collinearity, these methods can still be prone to failure as collinearity increases (Dormann et al., 2013). To reduce collinearity, the variance inflation factor (VIF) technique was used, regressing predictors against each other. A VIF value of 1 signifies no collinearity, with increasing values indicating greater collinearity. The optimal threshold for VIF is debated, though common rules of thumb are found in the literature (e.g., VIF = 10 is a commonly implemented cutoff) (O’brien, 2007). In this study, we adopted a conservative cutoff (VIF = 3) to minimize the potential impact of multicollinearity on model output. The resulting set of predictors used in baseline analyses, before further variable reduction procedures, were Annual Temperature (Bio1), Temperature Seasonality (Bio4), Mean Temperature of Wettest Quarter (Bio8), Precipitation of Wettest Month (Bio13), Precipitation Seasonality (Bio15), Elevation, Soil Organic Carbon Stock, and Soil Volumetric Water Content.

### Species distribution modeling

SDM for all *Oryzomys palustris*, eastern and western clades, was conducted over the contiguous United States using the BRT approach. Briefly, BRT modeling is a machine learning genetic algorithm that produces a boosted ensemble of simple regression trees for predicting species occurrence using environmental covariates and presence (i.e., species occurrences) and pseudo-absences (i.e., background points) (Elith et al., 2008). For BRTs, boosting is the process of developing an initial decision tree with inadequate predictive error (e.g., shallow tree) and then sequentially adding trees and their residuals together to improve predictive performance. Each tree ensemble is considered one model, which undergoes internal predictive evaluation by the *k*-fold cross-validation method that splits the occurrence dataset (i.e., presence and pseudo-absence localities) into fixed subsets utilized for model testing once, and training *k* − 1 times. An area under the receiver operator characteristics (AUC-ROC) curve is produced to demonstrate model predictive performance by exhibiting the true positive rate (sensitivity) and false positive rate (1 − specificity) of the model for predicting between presences (1) and pseudo-absences (0). Values of AUC exceeding 0.5 indicate that the model predicted presences better than random, with 1 being a perfect prediction. Furthermore, each model was evaluated using external and internal test data randomly selected from thinned presences and pseudo-absences prior to and during model development, respectively.

The selection of pseudo-absence locations (i.e., background data) impacts model development as much as species presence data. The use of target species for pseudo-absences is generally considered the optimal method, though a random selection of background data is acceptable when target species are not available (Cerasoli et al., 2017; Phillips et al., 2009; Stokland et al., 2011). It is suggested that the number of pseudo-absences should be approximately equal to the number of presence locations in BRT analyses (Barbet-Massin et al., 2012). Therefore, pseudo-absences (*n* = 400) were randomly selected for each model from a shapefile with buffered 10-km holes around each presence location (i.e., the Swiss-cheese method) to prevent known location resampling and for model convergence (Otieno et al., 2021). Replicate models were ensembled for averaging to further account for model stochasticity. These averaged models were then used to predict the probability of occurrence on the landscape.

The family of “gbm” functions in the R “dismo” package was used for BRT model production (“gbm. step”) and simplification (“gbm.simplify”). BRT simplification is backward elimination of low-contributing predictors using *k*-fold cross-validation (*n*-fold = 10) from an initial model, reducing the number of final model predictors. Fifty model replicates were generated for the simplification process, so predictors with the lowest average contribution were removed. Model replicate count and settings were preserved between the simplification step and final model processes. The “gbm” function incorporates numerous settings to customize model development, but the primary settings considered include model family, tree complexity (tc), learning rate (lr), bag fraction (bf), and maximum trees (mt). The “Bernoulli” family distribution was used because occurrence data were binary (i.e., presence and absence). Tree complexity refers to how many splits occur in a single regression tree, with each split considering increasing covariate interactions. At the same time, the learning rate determines the contribution amount of each tree to the ensembled model. Maximum trees generally reflect tree complexity and learning rate, as reducing these values requires additional trees to achieve model convergence. Bag fraction is the subset of training data randomly selected, without replacement, for tree construction, which implements stochastic gradient descent and reduces the opportunity for overfitting. Settings for the three SDMs were chosen to support model convergence and set as follows: tc = 3, lr = 0.0001, bf = 0.75, and mt = 100,000.

Relative variable influence (RVI) and partial dependency plots (PDPs) for predictor variables were used to visualize average variable contribution and relationships for model ensembles. The RVI is a measurement of whether a variable was selected in tree development and tree ensemble improvement (i.e., squared error decrease) through variable inclusion. All variables in the model contribute to tree construction, though some more than others. PDPs illustrate the marginal effects of the predictors on prediction outcomes of the model, including potential complex relationships between the predictors and response (Friedman, 2001; Molnar, 2020). The PDP curve for each variable is produced while holding the other variables at their average value, but can be skewed due to collinearity, which we reduced through the VIF process. The resulting RVIs and PDPs were averaged for each run of 50 ensembled models.

Averaged landscape-projected probabilities from each model run (i.e., full dataset, eastern clade, and western clade models) were recoded as binary geographic distributions to visualize and compare output across models. Rasters of continuous model output were dichotomized at three different probability thresholds of presence (0.5, 0.6, and 0.7) in ArcMap (ver 10.7.1), where pixels with probabilities exceeding a given threshold were recoded as present. Recoded distributions were combined using the “Raster Calculator” tool in ArcMap’s Spatial Analyst extension, allowing for visualization of range overlaps across models.

## RESULTS

**FIGURE 3:**
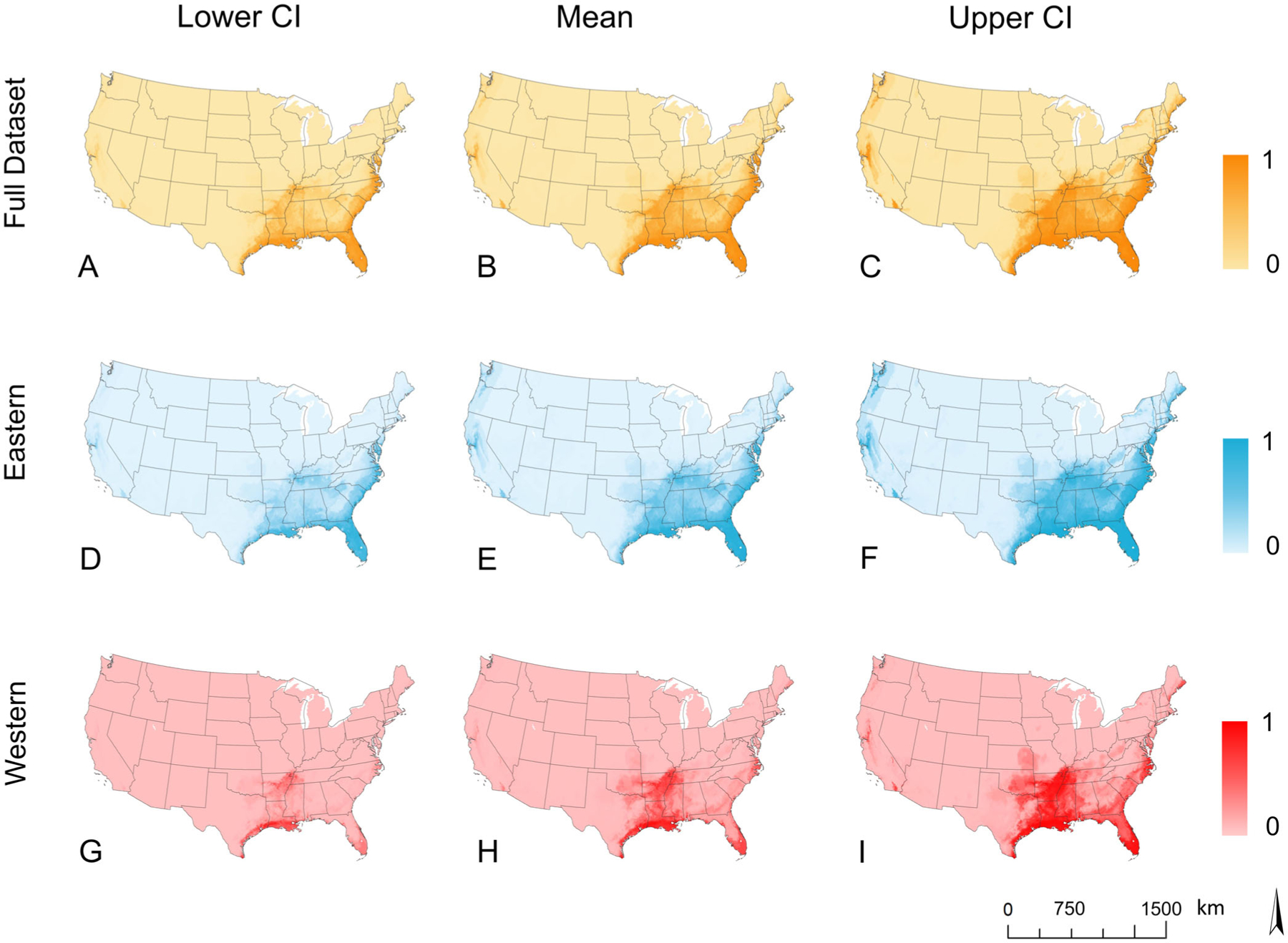
Potential distribution prediction of *Oryzomys palustris* full dataset (A–C), eastern clade (D–F), and western clade (G–I) occurrence in the United States based on the mean prediction of an ensemble of 50 boosted regression tree experiments including lower (2.5%; left) and upper (97.5%; right) confidence intervals.

The predicted geographic distribution of *O. palustris* primarily spans the southeastern United States and along the eastern Atlantic coastline. Mean landscape predictions for the three models (Figure 3B,E,H) primarily occurred in four US regions: the West Coast, the Southeast, the Gulf Coast, and the East Coast. The West Coast, including Washington, Oregon, and California, was primarily represented in the full dataset model and eastern clade model with maximum probabilities of 0.817 and 0.821, but the western clade model projected lower probabilities (maximum probability = 0.379) on the West Coast. The western clade model also predicted a smaller area of suitability on the West Coast than the other models. In all three models, the predicted Southeast range spanned Tennessee, Arkansas, Mississippi, Louisiana, Alabama, and Florida, extending north to the southern tip of Illinois and West Virginia, and extending west into Texas, Oklahoma, and Kansas. The full dataset (maximum probability = 0.834) and western clade (maximum probability = 0.826) models had the highest predicted suitable habitat in southeastern states compared with the eastern clade (maximum probability = 0.744). Although the western clade model prediction probability does reduce eastward, the other models maintain predictive consistency across the region. Maximum predicted probabilities for the full dataset (0.917) and eastern (0.916) models are the same for the Gulf Coast and East Coast regions. These models also have consistent prediction probabilities from the Gulf Coast of Texas to Maryland and taper off further north. The western clade model predicts the Texas and Louisiana sections of the Gulf Coast with a maximum probability of 0.928, but probabilities are not consistent towards Florida as in the other models, and the maximum probability for the East Coast is 0.708.

**FIGURE 4:**
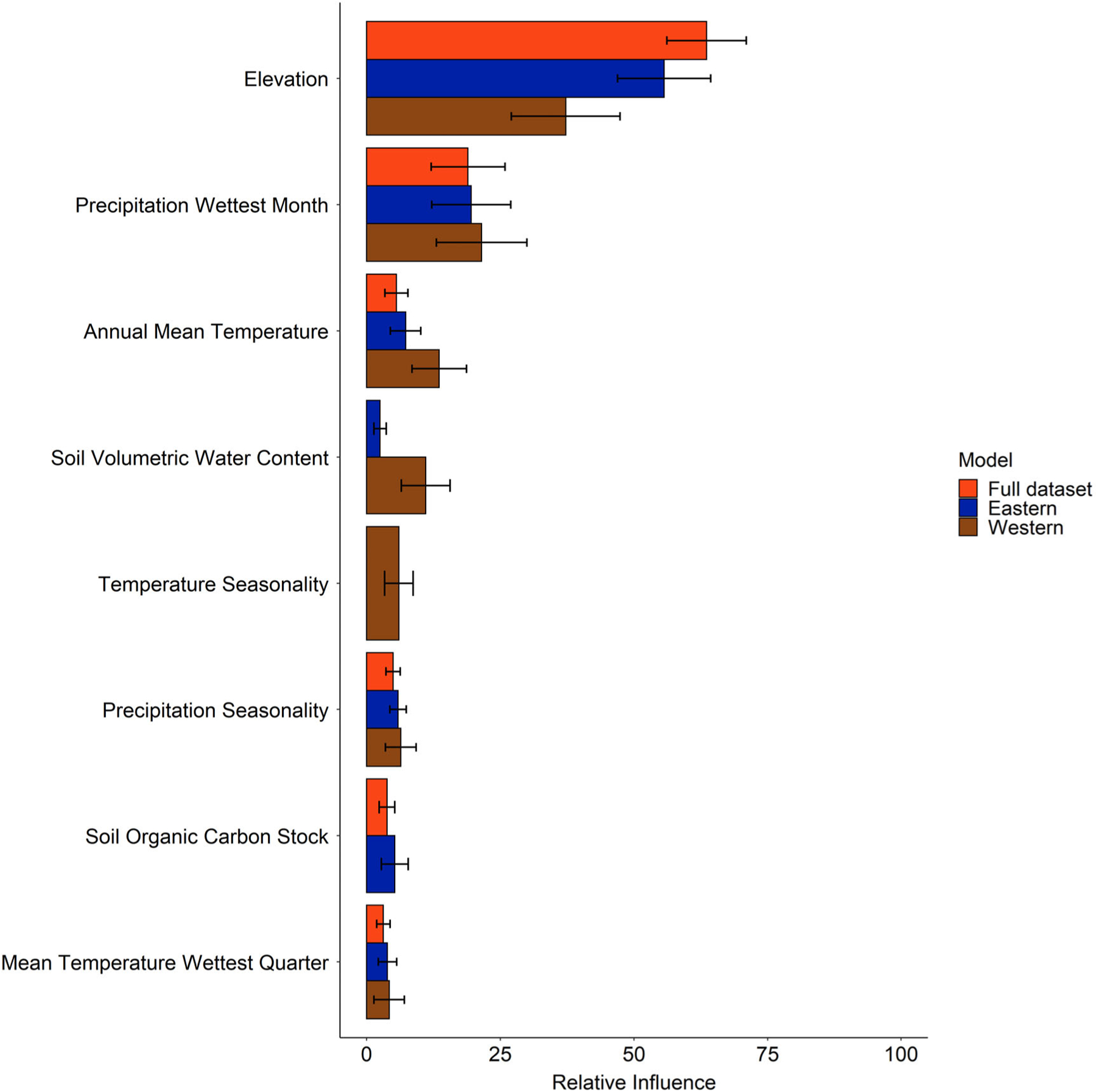
Variable relative influence for the final variable set used to model the distribution for the full *Oryzomys palustris* dataset, eastern clade, and western clade in the United States using boosted regression tree (BRT) experiments. Error bars represent variability across an ensemble of 50 BRT experiments.

**FIGURE 5:**
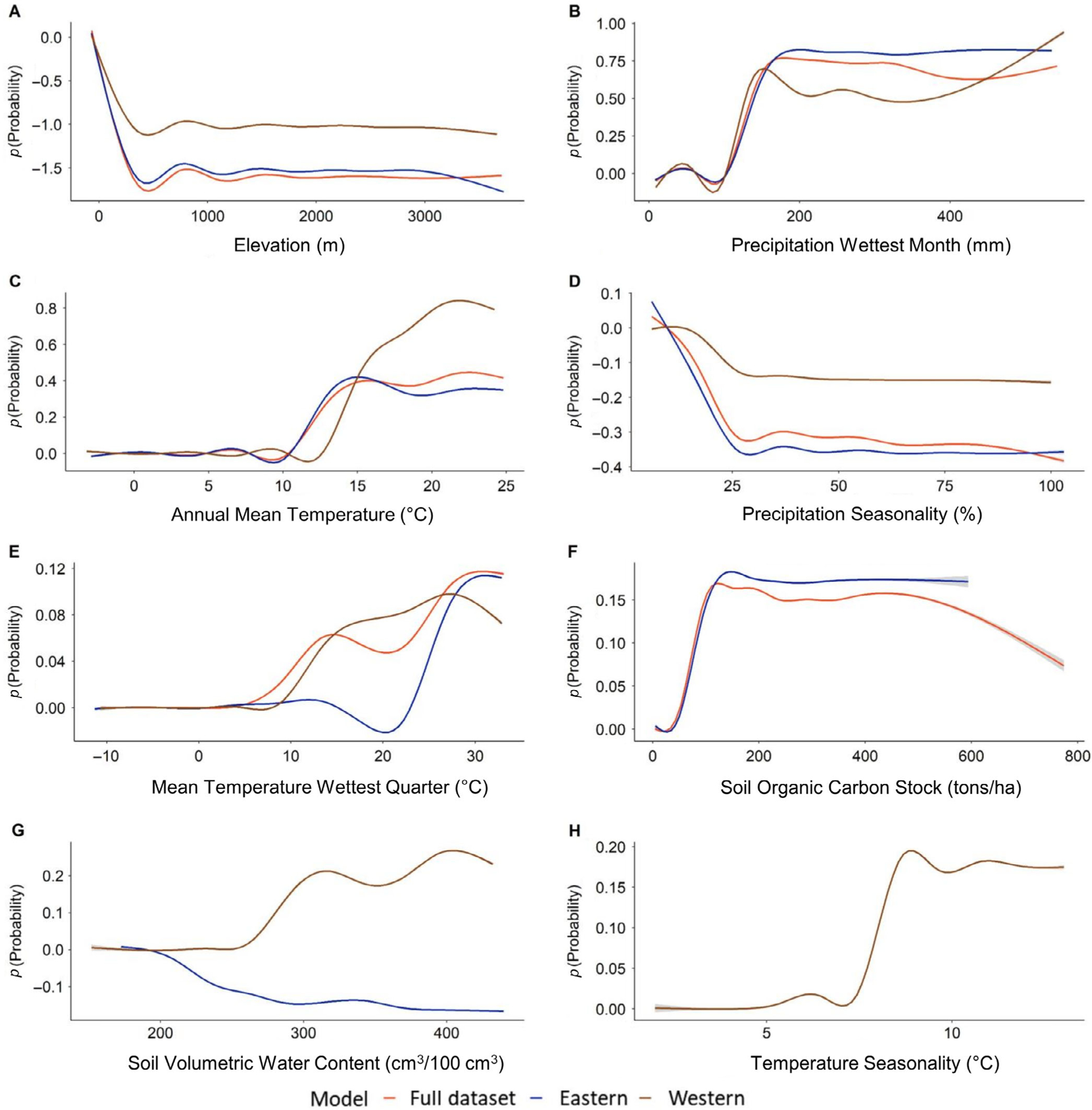
Partial dependency plots showing marginal effects on the prediction probability of the potential distribution of *Oryzomys palustris* models of the full dataset, eastern clade, and western clade by each variable across the 50 boosted regression tree experiments.

All three models had similar responses to the covariates consistent with occurrence data partitioning and overlap between models (Figure 4); however, differences in the magnitude of relationships between predicted probabilities and environmental predictors were apparent, especially between the western clade and the other two models (Figure 5). Elevation was the top contributing environmental predictor in all three models; prediction probability was highest at sea level and rapidly decreased with increasing elevation. The western clade model plateaued in prediction probability reduction earlier than the other models. Precipitation of the wettest month and annual mean temperature were the next highest contributors. All models generally exhibited positive relationships with increasing precipitation and temperature beginning around 100 mm and 10°C, respectively. There were some distinctions in the relationship with soil volumetric water content between the eastern and western models, as the former maintained a negative relationship while the latter had a positive relationship. However, this variable contributed minimally to the eastern model. The BRT simplification process excluded soil volumetric water content from the full dataset model and soil organic carbon stock from the western model (Figure 4). Temperature seasonality was excluded from the full dataset and eastern models (Figure 4).

All three models performed well with cross-validation, internal AUC values, and AUC values from external test data that remained above 0.9. The mean value for the full dataset model cross-validation AUC was 0.957 (SD = 0.007), internal test data AUC was 0.959 (SD = 0.015), and external test data AUC was 0.939 (SD = 0.005). The mean AUC of the eastern clade for cross-validation AUC was 0.960 (SD = 0.008), internal test AUC was 0.958 (SD = 0.013), and external test data AUC was 0.983 (SD = 0.004). The western clade model did have marginally higher standard deviations with AUCs of 0.965 (SD = 0.009) for cross-validation, 0.964 (SD = 0.018) for internal test data, and 0.989 (SD = 0.011) for external test data. The accuracy metrics for all three models were similar, although the AUC value for external testing data from the full dataset model was the lowest of the three models.

**FIGURE 6:**
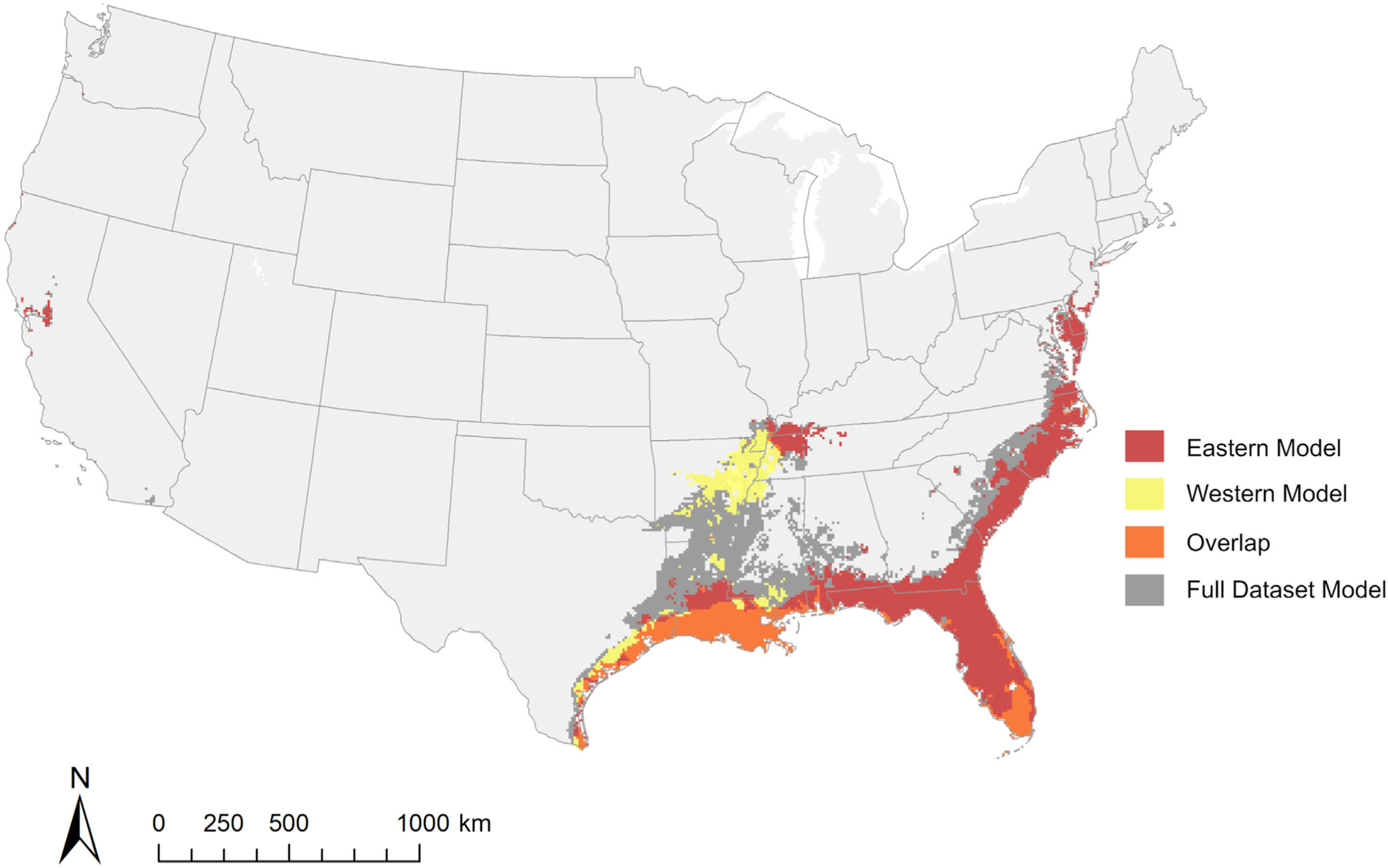
Overlap between mean dichotomized predictions of *Oryzomys palustris* suitability at a threshold for presence of 60% for models produced with the three datasets used in the study (i.e., full dataset, eastern clade, and western clade).

Dichotomized probabilities (at 0.5, 0.6, and 0.7) of mean landscape predictions for rice rat habitat suitability indicate the East Coast region extending from Maryland to Florida, peninsular Florida, the lower Mississippi River Basin, and the Gulf Coast region extending from Florida to Texas have the highest potential for rice rat species occurrence in all models (Figure 6; Appendix S1: Figures S1 and S2). The full dataset and eastern clade models predict occurrence across this entire area, with predictions of suitability extending further inland to Arkansas, Tennessee, Missouri, and Kentucky, and a small area on the West Coast (Figure 6; Appendix S1: Figures S1 and S2). Modeled distribution of the western clade is more restricted, by comparison, with predicted suitability primarily centered in Louisiana, Texas, and along the Mississippi River (Figure 6; Appendix S1: Figures S1 and S2). Distribution models for each type of data partitioning (full dataset, eastern clade, and western clade) overlap in mean prediction along the western portion of the Gulf Coast, primarily in Texas, Louisiana, and southern peninsular Florida (Figure 6; Appendix S1: Figures S1 and S2).

## DISCUSSION

This work is the first effort to apply SDM techniques to estimate the geographic distribution of the *O. palustris* species complex. The predicted distributions of all three taxonomic groupings were found to be predominantly restricted to coastal areas in the southeastern United States. Additionally, the estimated suitable habitat for both *O. palustris* taxonomic groupings (i.e., the full dataset and the eastern subset) extends into the southern portion of the Mid-Atlantic region, with a discontinuous, limited area of suitability in coastal California. In contrast, habitat suitability for the western clade is primarily highest along the Gulf Coast, extending west into coastal Louisiana and Texas. Further, output across models indicates a corridor of suitable habitat that extends north, throughout Louisiana, and into Arkansas, Mississippi, Tennessee, Kentucky, and southeastern Missouri, an area that coincides with the lower Mississippi River Basin. These spatial trends in estimated habitat suitability are perhaps unsurprising, as *O. palustris* is a semiaquatic rodent species that is primarily associated with both freshwater and estuarine wetlands, including tidal marshes, watersheds, and emergent wetlands (Eubanks et al., 2011; Kruchek, 2004). Rice rats are heavily dependent on the vegetation typically associated with coastal wetlands, including common wetland plants such as sedges (*Carex* spp.), rushes (*Juncus* spp.), cattails (*Typha* spp.), panic grass (*Panicum* spp.), and salt grass (*Spartina alterniflora*) (Eubanks et al., 2011; Rose & McGurk, 2006; Svihla, 1931). Suitable vegetation is a critical microhabitat feature for *O. palustris*, directly providing essential resources as both cover and food (Eubanks et al., 2011; Kruchek, 2004; Rose & McGurk, 2006). We were limited in this study by the inability to directly include environmental predictors related to land cover, as few *O. palustris* collections were made after the year 2000 in our datasets. Future work that ties contemporary rice rat observations with current land cover will enable us to assess reliance on vegetation type with greater resolution.

Elevation was the environmental predictor that contributed the most to estimated rice rat distributions, regardless of taxonomic clade. The highest probabilities of occurrence coincide with areas of low elevation at or near sea level, predominantly along coastlines in the Southeast and Gulf states. Precipitation of the wettest month and annual mean temperature were also influential factors in all three models, where higher precipitation values and temperatures exceeding ~13°C were associated with increasing habitat suitability. Together, elevation, precipitation, and temperature are likely capturing qualities of low-elevation marsh and wetland habitats that are necessary to maintain wetland plant communities and support rice rat populations. When considering other potential environmental drivers of rice rat populations, we start to see differences between the models for different taxonomic groupings. Low precipitation seasonality and soil organic carbon stock exceeding ~100 tons/ha are associated with increased probability of occurrence in the full *O. palustris* and eastern clade models. Again, these are factors that may support stable and robust plant communities in wetlands. In contrast, precipitation seasonality did not cause the same suitability drop-off in the western clade model. Instead, soil volumetric water content and temperature seasonality become important environmental predictors, indicating that water availability and temperature are more limiting factors for western rice rat populations.

Estimating the range of *O. palustris* is an important step forward in delineating the general distribution of potential hosts for *A*. *maculatum* and the rickettsial pathogens they transmit. Reservoir hosts involved in tick-borne transmission cycles are targets for control efforts and may serve as better indicators of risk than vector presence alone, particularly for ticks with widespread distributions. However, vertebrate hosts involved in many tick-borne transmission cycles are poorly understood or unknown. This is particularly true for SFG pathogens, many of which have only recently garnered increased scrutiny from medical and public health authorities, relative to other tick-borne diseases such as Lyme disease, where pathogen–host–vector dynamics are extensively studied (Halsey et al., 2018; Ostfeld, 2012). Components of the transmission cycle for *R. parkeri* are not currently known, but several small mammal species are suspected to play a role in the pathogen’s ecological maintenance, based on field surveys and laboratory experiments (Cumbie et al., 2020; Moraru et al., 2013). Investigation of the potential role of *O. palustris* as a reservoir host for *R. parkeri* is warranted. In addition to detection of *R. parkeri* in field-collected *O. palustris*, other closely related species of the genus *Oryzymys* have been established as competent hosts of rickettsial pathogens (Krawczak & Labruna, 2018; Padilha et al., 2013). Although the marsh rice rat has suspected involvement, further work is needed to establish the competency of *O. palustris* as a host capable of supporting the *R. parkeri* transmission cycle.

**FIGURE 7:**
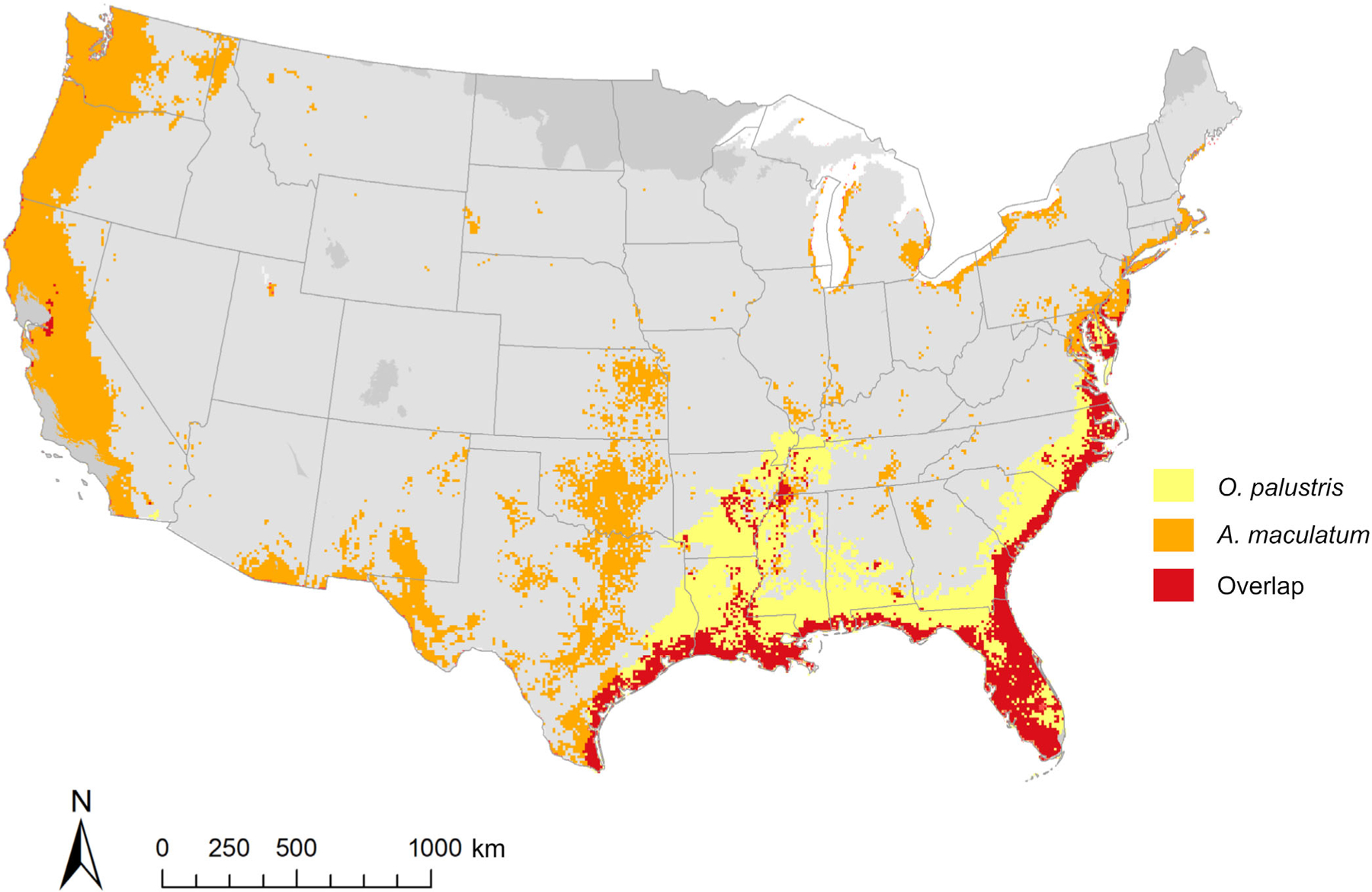
Overlap between mean dichotomized (threshold of 60%) full dataset model and predicted distribution *Amblyomma maculatum*, adapted from Flenniken et al. (2022).

There is considerable geographic overlap with *O. palustris* and *A. maculatum* in the southeastern United States. Comparing the model of our full *O. palustris* dataset to an SDM for *A. maculatum* produced by Flenniken et al. (2022), most of the overlap seen in estimated distributions occurs along the Gulf Coast, peninsular Florida, and along the East Coast into Virginia, Maryland, and New Jersey, with some incursion along the lower Mississippi River Basin (Figure 7; Appendix S1: Figures S3 and S4). The availability of suitable hosts may be an important element of successful range expansion of *A. maculatum*, which has drastically expanded the northern extent of its range in recent years (Molaei et al., 2021; Nadolny & Gaff, 2018; Wojan et al., 2022). Notably, much of this northward expansion has occurred along the East Coast, a potential indicator of the role that underlying host species like *O. palustris* play in the habitat suitability of tick vectors. The ranges of both *O. palustris* and *A. maculatum* also coincide with much of the estimated distribution of *R. parkeri* in North America, as described in Moo-Llanes et al. (2021). Moo-Llanes et al. built SDMs with records of ticks, comprised of several *Amblyomma* species, that tested positive for *R. parkeri* bacteria. The resulting model shows high predicted suitability in North America along the Gulf of Mexico and portions of the East Coast, with moderate to high predicted suitability extending inland throughout the eastern United States.

In addition to *O. palustris*, two other rodents in the family Cricetidae have also been implicated as hosts of *A. maculatum*, with potential involvement in the transmission cycle for *R. parkeri*: the hispid cotton rat (*Sigmodon hispidus*) and the eastern meadow vole (*Microtus pennsylvanicus*) (Cumbie et al., 2020). The geographic range of *S. hispidus* overlaps with much of our estimated distribution for *O. palustris*, extending further north into Nebraska and west into California, whereas the range of *M. pennsylvanicus* has some overlap with the northern extent of the *O. palustris* distributions, but extends north into Canada and Alaska (Jackson & Cook, 2020; USGS, 2018). Although *S. hispidus* has been documented as an important blood-meal host for immature *A. maculatum*, lab experiments have demonstrated that this species quickly clears *R. parkeri* infection, making them unlikely reservoir hosts (Baker et al., 2003; Johnson et al., 2022; Moraru et al., 2013). Likewise, *M. pennsylvanicus* is not a likely reservoir host of *R. parkeri*. While infected *A. maculatum* have been collected from *M. pennsylvanicus*, the ranges of the rodent and tick have minimal historical overlap (Cumbie et al., 2020; Nadolny & Gaff, 2018). Nevertheless, these three rodent species collectively span a vast area, potentially facilitating the expansion of ticks that utilize these species as hosts and providing opportunities for pathogen spillover events.

The underlying distribution of reservoir hosts is suspected to play a role in the geographic risk of tick-borne disease transmission. Tick-borne pathogens can exhibit geographically restricted ranges that are “nested” within the larger distribution of competent vectors. For example, within the eastern portion of the United States, foci for *Borrelia burgdorferi*, the spirochete that causes Lyme disease, are restricted within the range of its vectors, primarily *Ixodes scapularis* (Fleshman et al., 2021). The existence of an “infected” niche of ticks that tested positive for *R. montanensis* infections within the full geographic range of its vector, *Dermacentor variabilis*, has also been supported with SDMs (Lippi, Gaff, White, St. John, et al., 2021). The absence of pathogen transmission can be due to many factors, and certainly, vector presence alone is not sufficient for transmission. Absence of pathogens could be a result of undersampling or a lack of robust surveillance for tick-borne disease. Differences in suitable environmental conditions for tick presence and pathogen transmission may also contribute to range discrepancies (Galletti et al., 2016). However, the importance of reservoir hosts in maintaining vector-borne transmission cycles is widely acknowledged, yet in the case of many tick-borne diseases, not well understood. Even so, there is increasing evidence to support the key role that reservoir and amplifying hosts play in the localized density of infected ticks, and risk of transmission for an array of tick-borne diseases (Krawczyk et al., 2020). This has notably been the case with Lyme disease, where increasing burden and range expansion of the disease have been linked to range shifts and population changes in vertebrate hosts (Levi et al., 2012; Thompson et al., 2001).

## CONCLUSION

Conclusive identification of reservoir and amplifying hosts is still needed to establish transmission cycles for many tick-borne diseases, including the majority of those caused by rickettsial agents. Given that many species of medically important ticks have generalized habitat requirements, or are actively expanding their geographic ranges, identifying areas where tick presence overlaps with suitable reservoir habitat may be necessary for refining risk maps and public health advisories derived solely from tick presence. In this study, we estimated the geographic range of *O. palustris*, a suspected reservoir host of *R. parkeri*, and found considerable overlap with the distribution of *A. maculatum*. Though future work is needed to investigate the definitive role of *O. palustris* in transmission cycles, our findings highlight the potential need for extended surveillance efforts in those overlapping areas.

## Supplementary Material

Supplementary materials

## Figures and Tables

**TABLE 1:** Variables from WorldClim or the International Soil Reference and Information Center (ISRIC) considered for model development (MOD), including those removed due to collinearity through variance inflation factor (R).

Variable	Units	Source	Status
Annual Mean Temperature (Bio1)	°C	WorldClim ver 1.4	MOD
Temperature Seasonality (Bio4)	°C	WorldClim ver 1.4	MOD
Mean Temperature of Wettest Quarter (Bio8)	°C	WorldClim ver 1.4	MOD
Precipitation of Wettest Month (Bio13)	mm	WorldClim ver 1.4	MOD
Precipitation Seasonality (Bio15)	%	WorldClim ver 1.4	MOD
Elevation	m	WorldClim ver 2.1	MOD
Soil Organic Carbon Stock 30-cm depth	tons/ha	ISRIC	MOD
Soil Volumetric Water Content 33 kPa 0–5 cm depth	cm^3^/100 cm^3^	ISRIC	MOD
Maximum Temperature of Warmest Month (Bio5)	°C	WorldClim ver 1.4	R
Minimum Temperature of Coldest Month (Bio6)	°C	WorldClim ver 1.4	R
Temperature Annual Range (Bio7)	°C	WorldClim ver 1.4	R
Mean Temperature of Driest Quarter (Bio9)	°C	WorldClim ver 1.4	R
Mean Temperature of Warmest Quarter (Bio10)	°C	WorldClim ver 1.4	R
Mean Temperature of Coldest Quarter (Bio11)	°C	WorldClim ver 1.4	R
Annual Precipitation (Bio12)	mm	WorldClim ver 1.4	R
Precipitation of Driest Month (Bio14)	mm	WorldClim ver 1.4	R
Precipitation of Wettest Quarter (Bio16)	mm	WorldClim ver 1.4	R
Precipitation of Driest Quarter (Bio17)	mm	WorldClim ver 1.4	R
Precipitation of Warmest Quarter (Bio18)	mm	WorldClim ver 1.4	R
Precipitation of Coldest Quarter (Bio19)	mm	WorldClim ver 1.4	R

## Data Availability

The occurrence data used to build species distribution models in this study are openly available on the Global Biodiversity Information Facility (www.gbif.org), where we pulled North American records of *Oryzomys palustris* (https://doi.org/10.15468/dl.v9j4x5).
