# Supplementary material for: Estimating the distribution of Oryzomys palustris, a potential key host in expanding rickettsial tick-borne disease risk
Source: Ecosphere. Author manuscript; Available in PMC 2024 Aug 29. (PMC11359945; doi:10.1002/ecs2.4445)
Supplement: Supplementary materials [file NIHMS2005145-supplement-Supplementary_materials.pdf]

Ecosphere

Estimating the distribution of *Oryzomys palustris*, a potential key host in expanding rickettsial tick-borne disease risk

Catherine A. Lippi, Samuel Canfield, Christina Espada, Holly D. Gaff, and Sadie J. Ryan

Appendix S1

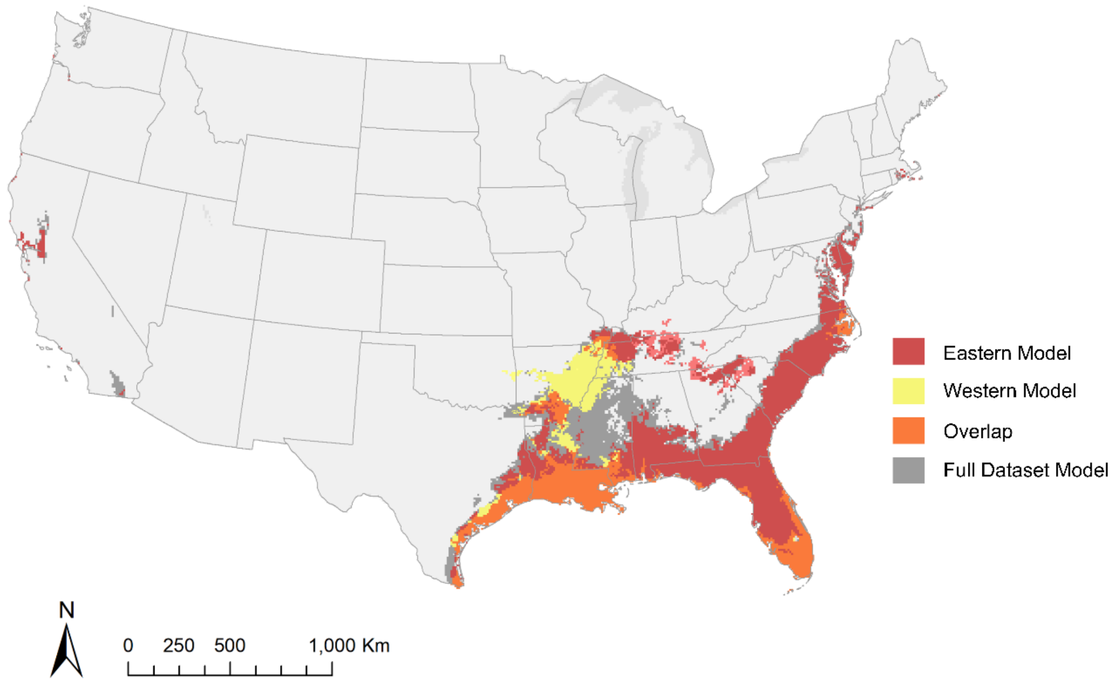

Fig. S1. Overlap between mean dichotomized predictions of *Oryzomys palustris* suitability at a threshold for presence of 50% for models produced with the three datasets used in the study (i.e., full, Eastern, and Western).

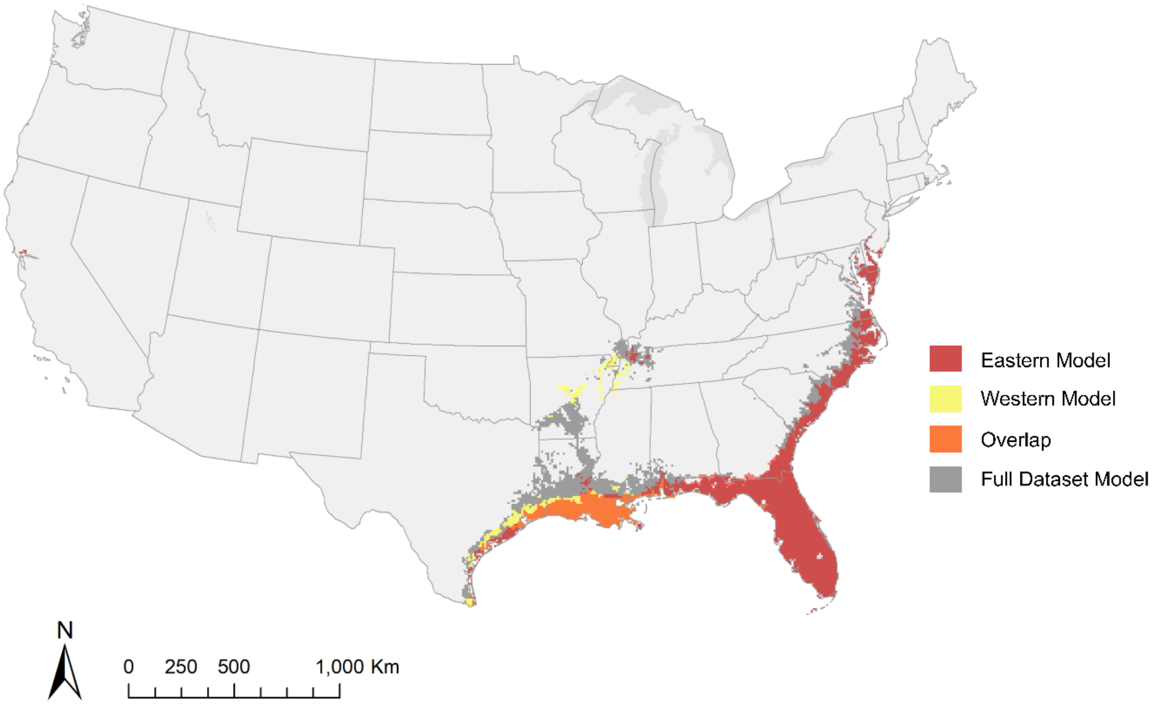

Fig. S2. Overlap between mean dichotomized predictions of *Oryzomys palustris* suitability at a threshold for presence of 70% for models produced with the three datasets used in the study (i.e., full, Eastern, and Western).

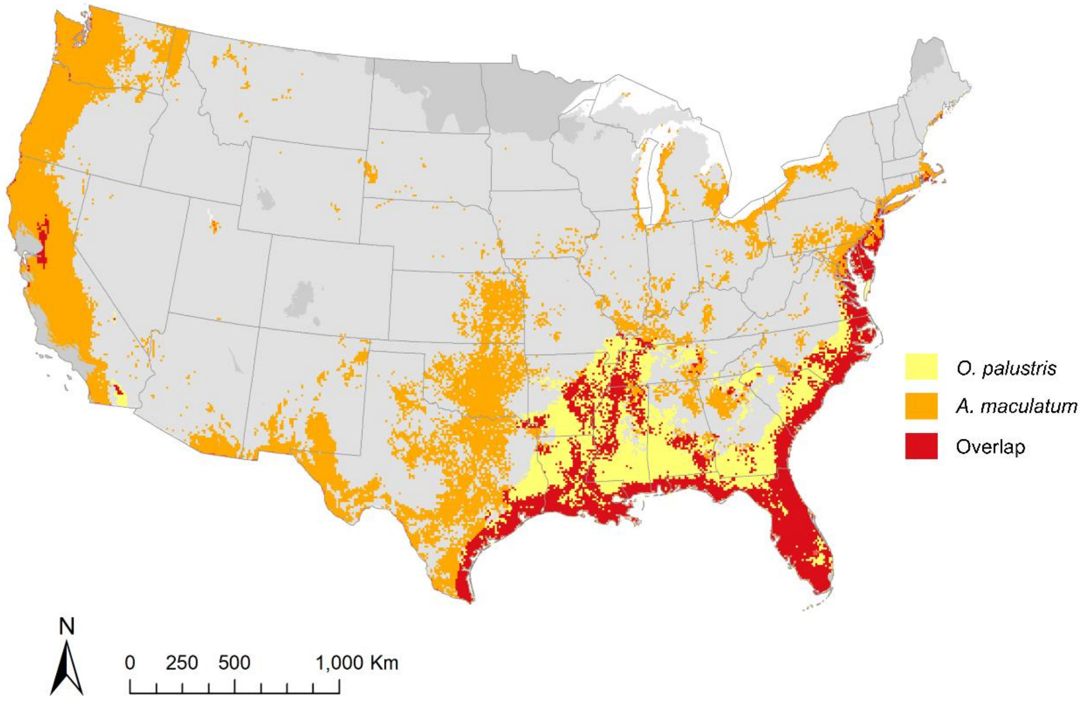

Fig. S3. Overlap between mean dichotomized (threshold of 50%) Full dataset model and predicted distribution *Amblyomma maculatum* from Flenniken et al. 2022.

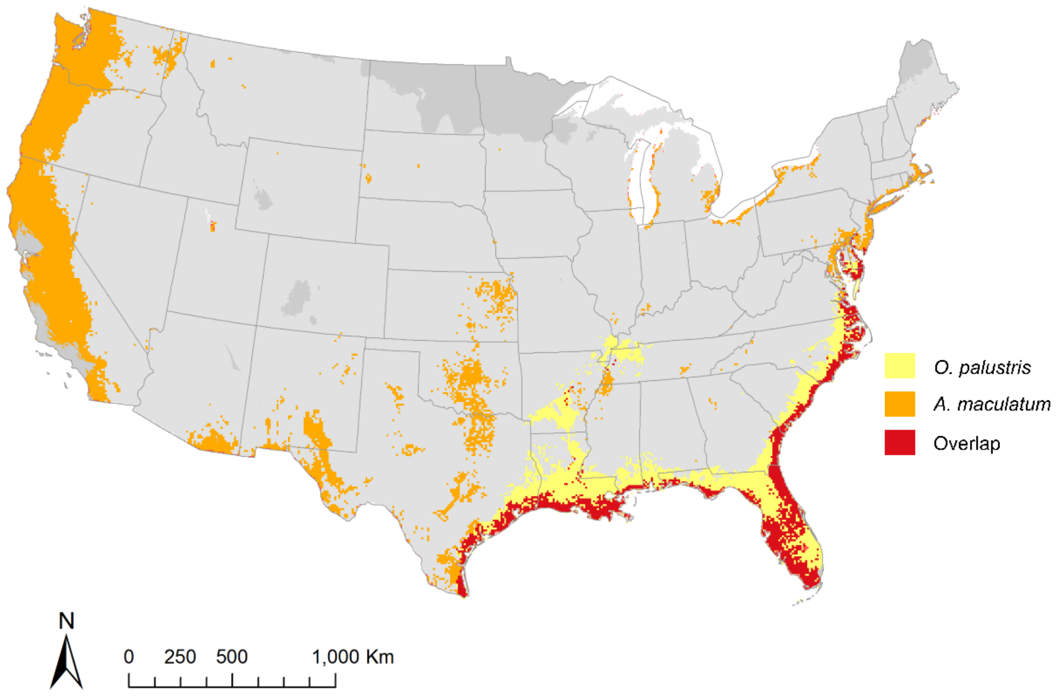

Fig. S4. Overlap between mean dichotomized (threshold of 70%) Full dataset model and predicted distribution *Amblyomma maculatum* from Flenniken et al. 2022.

Literature Cited

Flenniken, J. M., H. C. Tuten, H. Rose Vineer, V. C. Phillips, C. M. Stone, and B. F. Allan. 2022. Environmental Drivers of Gulf Coast Tick (Acari: Ixodidae) Range Expansion in the United States. Journal of Medical Entomology 59(5): 1625-1635.
